# Acute Kidney Failure in a Young African American Male

**DOI:** 10.1155/2019/2591560

**Published:** 2019-02-17

**Authors:** Thuan V. Nguyen, Nada M. Bader, Harpreet Sidhu, Caprice Cadacio, Susana M. Mendoza, Phuong Thu T. Pham, Phuong Chi T. Pham

**Affiliations:** ^1^Olive View-UCLA Medical Center, Division of Nephrology and Hypertension, Los Angeles, CA 91342, USA; ^2^David Geffen School of Medicine at UCLA, Kidney Transplant, Los Angeles, CA 90095, USA

## Abstract

Retroperitoneal fibrosis (RPF) is a condition characterized by chronic inflammatory and fibrotic changes in the retroperitoneum that can lead to serious complications including kidney failure, mesenteric and limb ischemia, and deep venous thrombosis among others. Affected individuals may present with nonspecific symptomology that would require a high clinical index of suspicion for prompt diagnosis. We herein discuss a case of a young African-American man with recurrent deep venous thrombosis who presents with a 4-week history of constant aching pain of abdomen and back and kidney failure. Initial noncontrast computed tomogram (CT) only revealed mild bilateral hydroureteronephrosis with inflammatory changes but without obvious mass or lymphadenopathy. At the insistence of the renal consulting team to rule out RPF, a CT-urogram was performed which revealed an infiltrative mass encasing the aorta, inferior vena cava, and common iliac vessels. Laparoscopic biopsy revealed dense fibroadipose tissue, lymphocytic aggregates, focal scattered IgG4-positive plasma cells, and fibrin deposition. Patient underwent bilateral nephrostomy placement and empirical corticosteroid therapy with resolution of kidney failure. Our case illustrates a classic presentation of RPF with relatively benign findings on noncontrast CT that could have been missed if clinicians did not keep a high index of suspicion for the condition.

## 1. Introduction

Idiopathic retroperitoneal fibrosis (RPF) is a relatively uncommon condition characterized by chronic inflammatory and fibrotic changes in the retroperitoneum that can lead to diffuse encasement and compression of retroperitoneal structures and a myriad of associated complications. Clinical symptomology ranges from nonspecific constitutional symptoms, vague diffuse abdominal or back pain, abdominal distension, and lower extremity edema to more serious complications including kidney failure, mesenteric and limb ischemia, and deep venous thrombosis, among others [1–3]. Prompt diagnosis of RPF may be challenging due to its nonspecific and broad clinical manifestations.

In patients who present with kidney failure from obstructive nephropathy, prompt diagnosis may be further hindered by the absence of significant hydronephrosis. It is plausible that compression of the kidneys, aorta, and renal arteries by inflammatory and fibrosing tissues leads to poor renal blood flow and low urine production, thus the appearance of relatively mild hydroureteronephrosis. Additionally, the level of RPF involvement may be more distal, which leads to hydroureters rather than overt hydronephrosis in early disease. Alternatively, kidney failure in patients with RPF may partly arise from renal ischemia due to the encasement of renal vasculature, in which case, hydronephrosis would not be observed. Finally, in the case of RPF associated with IgG4 related disease, kidney injury may be due to direct IgG4 related sclerosing interstitial nephritis independent of RPF-associated structural compromise [4].

We herein discuss a case of a young African American male who presents with relatively acute kidney failure of unknown etiology whose initial kidney ultrasound and noncontrast computed tomogram (CT) of abdomen and pelvis only revealed mild bilateral hydroureteronephrosis.

## 2. Case Report

*Clinical History.* A 27-year-old African American male with a history of unprovoked recurrent right lower extremity deep vein thrombosis and bilateral testicular hydrocele presented with a 4-week history of bilateral flank and generalized nonradiating lower abdominal pain. The pain was described as constant ache, associated with early satiety, but without nausea/vomiting, diarrhea, melena, or hematochezia. He denied weight loss or any urinary symptoms. Social history was negative for tobacco smoking and alcohol or recreational drug use. Medications included diphenhydramine as needed for sleep and apixaban.

*Physical Exam.* Vital vital signs revealed temperature 37.1°C, blood pressure 121/69 mmHg, pulse 96 beats/minute, and respiratory rate 16/minute. Lungs and heart exams were unremarkable. Lower half abdomen was mildly tender with palpation, but without mass, rebound, or guarding. There was mild right costovertebral angle tenderness and trace bilateral pedal edema. Genitalia were within normal limits without edema.

**Figure 1:**
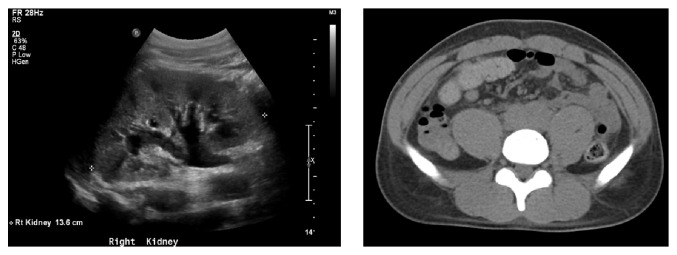
Initial imaging studies. Left: renal ultrasound at presentation revealed moderately enlarged kidneys with increased renal cortical echogenicity and mild bilateral hydronephrosis. Right: abdomen and pelvis computed tomogram without contrast revealed mild inflammatory changes without lymphadenopathy.

*Initial Laboratory Data.* Routine chemistry revealed serum sodium 133 mEq/L, potassium 5.0 mEq/L, chloride 98 mEq/L, bicarbonate 25 mEq/L, blood urea nitrogen 57 mg/dL, creatinine 12.6 mg/dL (baseline of 1.0 mg/dL one month prior), estimated glomerular filtration rate 6 mL/min/1.73 m^2^, and calcium 8.6 mg/dL. Urinalysis revealed specific gravity 1.014, pH 8.0, no red or white blood cells, 100 mg/dL protein, and no glucose or blood. Urine protein to creatinine and albumin to creatinine ratios were 0.5 and 0.293 g/g creatinine respectively. Renal ultrasound and abdomen/pelvis CT without contrast revealed mild bilateral hydroureteronephrosis with nonspecific inflammatory changes (Figure 1).

The bland urinalysis and lack of significant proteinuria/albuminuria suggest tubulointerstitial injury (late phase of acute tubular necrosis, chronic tubulointerstitial nephritis) or obstructive uropathy. Differential diagnoses of obstructive uropathy relevant to current African American patient with unknown sickle cell history include recently passed papillary necrotic tissues or bladder stone, complicated medullary carcinoma (associated with sickle cell trait), diphenhydramine-induced neurogenic bladder, or retroperitoneal fibrosis (RPF). Rapidly progressive glomerulonephritis is thought to be less likely given the bland urinalysis.

*Additional Investigations.* Given the mismatched relatively benign findings of both urinalyses and imaging studies and degree of kidney failure and associated symptomology suspicious for RPF, an abdomen and pelvis CT with intravenous contrast was obtained.

**Figure 2:**
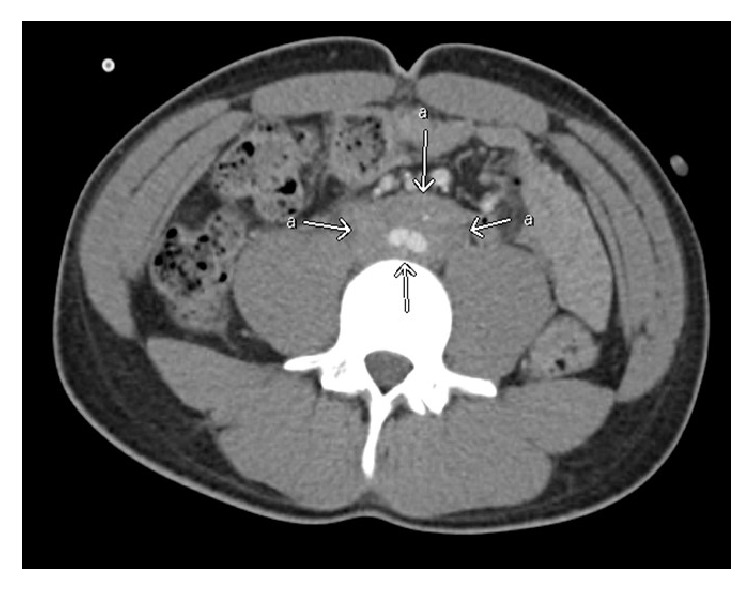
Abdomen and pelvis computed tomogram with intravenous contrast revealed mild bilateral hydroureteronephrosis with point of obstruction at the level of distal abdominal aorta and inferior vena cava. There was an ill-defined, infiltrative soft tissue mass encasing both aorta and IVC (approximately 7.2 cm). The mass appeared to infiltrate along the bilateral proximal common iliac vessels.

Abdomen and pelvis computed tomogram with intravenous contrast revealed mild bilateral hydroureteronephrosis with point of obstruction at the level of distal abdominal aorta and inferior vena cava. There was an ill-defined, infiltrative soft tissue mass encasing both aorta, and inferior vena cava (approximately 7.2 cm). The mass appeared to infiltrate along the bilateral proximal common iliac vessels (Figure 2).

Serologies to evaluate for autoimmune diseases and infectious etiologies including C-reactive-protein, human-immunodeficiency-virus, QuantiFERON gold, and antinuclear-antibody were negative. Serum lactate dehydrogenase obtained for possible lymphoproliferative disorder was mildly elevated at 248 IU (reference <192 IU). IgG4 level was 44.2 mg/dL (reference 4-86 mg/dL). Laparoscopic retroperitoneal mass biopsy revealed dense fibroadipose tissue with lymphocytic aggregates, focal scattered IgG4 positive plasma cells, and fibrin deposition without malignant cells.

*Diagnoses.* The diagnosis of idiopathic RPF was made. Kidney failure was thought to be due to severe bilateral ureteral encasement by RPF.

**Figure 3:**
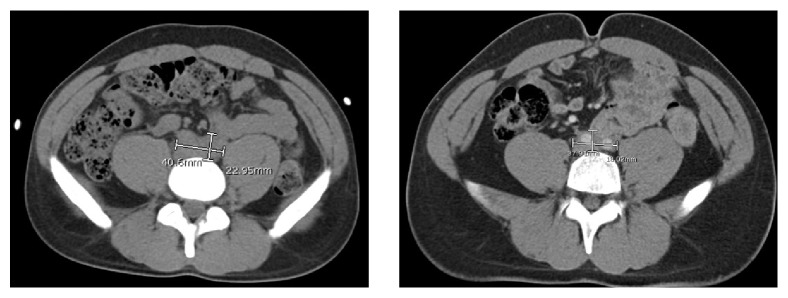
Follow-up abdomen and pelvis computed tomogram with and without intravenous contrast 4 months following a course of corticosteroid therapy revealed significant reduction in the soft tissue mass (approximately 4 cm in diameter compared with 7 cm at presentation).

*Clinical Follow-Up*. Patient underwent bilateral nephrostomy placement with rapid improvement of serum creatinine. In addition, patient received a trial of prednisone 40 mg daily. At 4-month follow-up, kidney function normalized and CT revealed marked reduction in RPF size (Figure 3).

## 3. Discussion

Retroperitoneal fibrosis is thought to be a rare condition with an estimated incidence of 0.1-1.3 cases/100,000 persons/year and prevalence of 1.4 per 100,000 persons. It is plausible that the true incidence and prevalence of RPF may be higher due to the clinicians' generally low index of suspicion for the diagnosis as RPF is a condition with very nonspecific signs and symptoms. Reported cases have been generally observed in individuals between the ages of 40 and 60 with a male predominance of 2:1-3:1 [1–3].

RPF may be associated with various autoimmune diseases including small-vessel vasculitis, rheumatoid arthritis, Sjogren's disease, and thyroiditis [1–3, 5] or linked to a number of presumed etiologic factors including drugs, malignancy, infections, radiation, and trauma (Table 1) [2, 6–8]. In most cases, however, RPF is idiopathic where the inciting agent or event remains elusive [2]. The common pathway leading to RPF is not known but suggested to be via an exogenous antigen-driven or autoimmune process. Genetic polymorphisms (e.g., HLA-DRB1*∗*03, ∆32 polymorphism of the gene encoding CCR5, a chemokine receptor, and TTCCAT haplotype of the gene encoding CCL11/eotaxin-1) and environmental exposures to tobacco smoking and asbestos are thought to be predisposing factors to the development of RPF [1, 9]. The underlying pathogenesis of RPF has been suggested to be antigen-driven, where antigen presenting cells activate CD4+ T-lymphocyte to produce interleukin-6 (IL-6), thereby leading to B-cell and fibroblast activation. The secretion of other interleukins including IL-4, IL-10, and IL-13 by activated CD4+ T-lymphocytes further lead to B-cell expansion and maturation into plasma cells, presumably with a preferential expansion of IgG4-producing plasma cells. Lymphoid cells may further secrete eotaxin-1, which effectively recruits eosinophils and mast cells to affected areas where they in turn produce cytokines to activate fibroblasts. Activated fibroblasts subsequently mature into myofibroblasts and secrete collagen to form fibrosing tissues (reviewed in [1]). As IgG4+ plasma cells are observed in both idiopathic RPF and IgG4-related disease (IgG4-RD), RPF has been recently suggested to be part of the spectrum of IgG4-RD [10–12].

Clinical manifestations reflect the underlying inflammatory process and dysfunction of affected structures, Table 1 [1–3, 6–9]. Most common manifestations appear to be flank and abdominal pain and lower extremity edema. Others include nonspecific abdominal distension, fatigue and sleepiness, nausea, low grade fevers, lower extremity and intestinal claudication, deep venous thrombosis, urinary symptoms such as frequency, dysuria, and hematuria, and hypertension. Although rare, intestinal infarction has been reported [13]. Testicular manifestations may occur in up to 50% of reported cases and may manifest as testicular pain, with or without hydrocele, varicocele, or both, presumably due to RPF encasement of the spermatic vein, retrograde ejaculation, and erectile dysfunction [1–3, 6–9]. It is plausible that male genitourinary manifestations may lead more male patients to seek medical care, thus higher diagnosis rate among males compared with that of females, rather than the disease having a predilection for males. Laboratory findings may reveal kidney injury in 40% to 70% and inflammatory markers including C-reactive protein and erythrocyte sedimentation rate in 50% to over 80% of cases [1–3, 6–8]. Serological markers including serum IgG4 and antinuclear antigen may be positive in over 25% of patients [8].

Histologically, RPF consists of inflammatory infiltrates, typically involving lymphocytes, plasma cells, and macrophages that are interspersed within collagen type I bundles or organized into nodular aggregates encircling small retroperitoneal vessels. RPF may be considered part of IgG4-related disease when plasma cells predominate inflammatory infiltrates, with IgG4+ to total IgG+ plasma cell ratio exceeding 40% [10–12].

The diagnosis of RPF, particularly in the setting of kidney injury, relies on the clinicians' prompt recognition of its classic symptomology as initial low-quality imaging studies (e.g., routine kidney ultrasound evaluation for acute kidney injury) may be unimpressive relative to the degree of kidney failure. Consideration for possible RPF should be given in cases of unexplained lower extremity edema or claudication, recurrent deep venous thrombosis, vague abdominal or flank pain, or even scrotal pain/edema, particularly in cases where a constellation of these symptoms are concurrently present. CT with intravenous contrast or magnetic resonance imaging may be used to diagnose RPF. RPF typically appears as a homogeneous mass encasing various retroperitoneal structures including the anterolateral sides of the abdominal aorta common iliac arteries, ureters, and inferior vena cava [1, 3, 7, 8]. The use of ^18^F-fluorodeoxyglucose (^18^F-FDG) positron emission tomography (PET) has recently been suggested to be a beneficial tool in monitoring RPF activity and guiding ongoing therapy. ^18^F-FDG PET testing, however, is not specific for RPF and should not be used as a diagnostic tool [14]. Definitive diagnosis of the underlying etiology requires tissue biopsy and full evaluation for underlying malignancy, autoimmune diseases, and infectious etiology.

Management involves symptomatic relief for affected organs such as relief of urinary obstruction via nephrostomy placement as needed for kidney failure and anticoagulation therapy for deep venous thrombosis. Whenever applicable, treatment of the underlying etiology or withdrawal of offending agents and exacerbating factors should be considered. Once infectious and malignant sources have been ruled out, a trial of immunosuppressive therapy may be initiated. Corticosteroid is currently the mainstay of therapy. There is no consensus on the optimal corticosteroid regimen, but empirical therapy has been suggested to be prednisone 0.5-1.0 mg/kg/day with gradual taper to 5.0-7.5 mg/day within 6-9 months [8, 15]. Of note, however, relapse rates with corticosteroid monotherapy have been reported to be high and ranging from 9 up to 66% within a follow-up of 18 to 66 months [15]. Alternative therapeutic options including cyclophosphamide, tamoxifen, calcineurin inhibitors, azathioprine, among others for resistant or steroid-intolerant cases are listed in Table 2 [13, 16–23]. Given the reported high rate of relapse, close monitoring of disease activity is highly suggested.

## 4. Conclusions

In summary, RPF is presumably an uncommon and insidious disease that typically presents with nonspecific signs and symptoms. A strong clinical index of suspicion for RPF should be raised when patients present with an array of nonspecific signs and symptoms involving various structures from the abdomen and flank downward. Delayed diagnosis may lead to serious complications including kidney failure, deep venous thrombosis, bowel and extremity claudication, and even intestinal infarction. In patients with kidney involvement, the degree of kidney failure may be out of proportion to the presenting hydronephrosis. Awareness of this disease entity among clinicians is necessary to ensure prompt diagnosis and optimal outcomes. The mainstay of therapy involves the empirical use of corticosteroids but relapse rates are high with monotherapy. Close follow-up and addition of a second immunosuppressive agent may be required.

## Figures and Tables

**Table 1:** Etiologic factors and clinical manifestations of retroperitoneal fibrosis.

**Etiologic factors**	

Drugs	Methysergide and other ergot alkaloids, and less commonly, beta blockers, methyldopa, hydralazine, analgesics, etanercept, infliximab
Malignancy	Lymphoma, sarcoma, carcinoid, metastatic disease from primary cancers [e.g. gastrointestinal, breast, lung, genitourinary tract, thyroid])
Proliferative/infiltrative diseases	Erdheim-Chester disease, other histiocytosis, IgG4-related diseases
Infections	Mycobacterium tuberculosis, histoplasmosis, actinomycosis
Others	Radiation, asbestos exposure, trauma, idiopathic
**Clinical manifestations**	

Constitutional symptoms (as a result of underlying disease or consequences of any complications above)	Mild fevers, fatigue, sleepiness, anorexia, hypertension
Circulatory system	Aorta and its branches: Limb ischemia, claudication, testicular pain
	Inferior vena cava: Lower extremity deep venous thrombosis/phlebitis, lower extremity edema, varicocele
Gastrointestinal system	Pancreas: Pancreatitis (may be associated with underlying etiology of RPF, i.e. IgG4-related disease)
	Intestines (duodenum, ascending and descending colong): Abdominal pain (typically back, nonspecific to lower abdomen), abdominal distension, nausea, weight loss
Urinary tract	Adrenals: Fatigue, malaise
	Kidneys, ureters: Oliguria, anuria, kidney failure
Lymphatic system	Lumbar lymph nodes: Lower extremity edema, testicular hydrocele

**Table 2:** Alternative therapeutic options for retroperitoneal fibrosis.

Source	Study	n	Study Patients	Outcome
Vaglio et al., Lancet, 2011	Prednisone vs. tamoxifen (0.5 mg/kg/d) for 8 months	36	Patients in remission after 1 month of prednisone	Lower relapse rate in prednisone (17%) vs. tamoxifen (50%) group (p-value 0.04)

Van Bommel et al., Am J Kidney Dis, 2007	Prednisone (60 mg/d for 6 wks, tapered for 3 months to 10 mg/d for 1 y)	24	Patients with first presentation of idiopathic RPF	High initial success rate (75%) with high recurrence rate (72% of successes)

Van Bommel et al., Ann Intern Med, 2006	Tamoxifen (20 mg bid)	19	37% idiopathic RPF; 63% secondary to atherosclerosis	79% reported symptom improvement with 6% recurrence

Jois et al., Rheumatology, 2007	Cyclophosphamide (1 g/2 wks, 6 pulses) plus mycophenolate mofetil [MMF], 2.5 g/d) with reduced prednisolone	1	Relapsing RPF previously treated with reduced dose prednisolone and methotrexate	Asymptomatic No recurrence at 15 months

Scheel et al., Ann Intern Med, 2011	MMF (1g twice daily for 10-30 m) with prednisone(40 mg/d, tapered over 6 m)	7	1 patient had known RPF risk factor (external beam radiation)	Idiopathic RPF patients had 16%-62% mass regression; 10/11 ureters not obstructed after stent removal

Binder et al., Ann Rheum Dis, 2012	Cyclophosphamide plus low dose corticosteroid induction; long term azathioprine or methotrexate	35	All patients had chronic periaortitis	Significant reduction of transverse diameter of periaortic mantle Less time between insertion and removal of ureteral stents

Marcolongo et al., Am J Med, 2004	Prednisone plus either azathioprine or cyclophosphamide for 6 m	26	All patients had idiopathic RPF	Treatment failure rate of ~1 per 100 patient-years (95% CI: 0.02 to 5 per 100 patient-years)

Alberici et al., Ann Rheum Dis, 2012	Methotrexate (15-20 mg/wk) plus prednisone	14	All patients had relapsing idiopathic RPF	79% in remission, fewer relapses in patients continuing treatment; Significant CRP and ESR reduction, no significant mean mass reduction

Maritati et al., Ann Rheum Dis, 2012	Rituximab (375 mg/m^2^/wk for 4 wks)	2	Both patients had chronic periaortitis. One relapsing; other administered rituximab plus low dose prednisone	Remission achieved in both patients

n, number of patients; RPF, retroperitoneal fibrosis; MMF, mycophenolate mofetil; CI, confidence interval; CRP, C-reactive protein; ESR, erythrocyte sedimentation rate.
